# Large Intelligent Surfaces Communicating Through Massive MIMO Rayleigh Fading Channels

**DOI:** 10.3390/s20226679

**Published:** 2020-11-22

**Authors:** Ricardo Coelho Ferreira, Michelle S. P. Facina, Felipe A. P. de Figueiredo, Gustavo Fraidenraich, Eduardo Rodrigues de Lima

**Affiliations:** 1 DECOM/FEEC, State University of Campinas, Av. Albert Einstein 400, Campinas 13083-970, Brazil; rcoeferreira@gmail.com (R.C.F.); michelle.facina@gmail.com (M.S.P.F.); 2 Instituto Nacional de Telecomunicações, Santa Rita do Sapucaí 37540-000, Brazil; zz4fap@gmail.com; 3 Department of Hardware Design, Instituto de Pesquisas Eldorado, Campinas 3083-898, Brazil; eduardo.lima@eldorado.org.br

**Keywords:** large intelligent surfaces, massive MIMO systems, maximum ratio transmission, Von Mises distribution, Rayleigh fading

## Abstract

Large intelligent surfaces (LIS) promises not only to improve the signal to noise ratio, and spectral efficiency but also to reduce the energy consumption during the transmission. We consider a base station equipped with an antenna array using the maximum ratio transmission (MRT), and a large reflector array sending signals to a single user. Each subchannel is affected by the Rayleigh flat fading, and the reflecting elements perform non-perfect phase correction which introduces a Von Mises distributed phase error. Based on the central limit theorem (CLT), we conclude that the overall channel has an equivalent Gamma fading whose parameters are derived from the moments of the channel fading between the antenna array and LIS, and also from the LIS to the single user. Assuming that the equivalent channel can be modeled as a Gamma distribution, we propose very accurate closed-form expressions for the bit error probability and a very tight upper bound. For the case where the LIS is not able to perform perfect phase cancellation, that is, under phase errors, it is possible to analyze the system performance considering the analytical approximations and the simulated results obtained using the well known Monte Carlo method. The analytical expressions for the parameters of the Gamma distribution are very difficult to be obtained due to the complexity of the nonlinear transformations of random variables with non-zero mean and correlated terms. Even with perfect phase cancellation, all the fading coefficients are complex due to the link between the user and the base station that is not neglected in this paper.

## 1. Introduction

The future of mobile digital communications in the age of the internet of things (IoT) requires to optimize the energy consumption for transmission, improve the signal to noise ratio (SNR) at the receiver, increase the spectral efficiency, and propose communication protocols, channel estimation methods and beamforming strategies suitable for the adopted system model.

Many solutions have been proposed as alternatives to the sixth generation of mobile communications. Zhang et al. [1] make an excellent review of the literature on these emerging techniques, citing among them large intelligent surfaces (LIS), holographic beamforming (HBF), angular orbital momentum (OAM) multiplexing, laser and visible-light communications (VLC) [2] and the advent of quantum computing which is increasingly present in large technology companies like Google and allows unmatched performance and security for quantum communication systems. Nawaz et al. [3] investigate the use of quantum machine learning strategies to improve the performance of the processes involved in the network structure since we mess with many parallel operations involving large arrays and tensors with data loaded and that through quantum computing can be mapped into large tensors product spaces where operations are handled by quantum processors that take advantage of the phenomenon of quantum superposition to achieve large communication rate and encryption security.

The proposal to make use of large intelligent surfaces (LIS) to improve transmission quality in massive MIMO systems is recent and has been gaining visibility in the literature as a concrete solution for the sixth generation (6G) of mobile communications and has presented a competitive performance in comparison with classic methods like relaying switches.

One of the great challenges for the implementation of the LIS is to estimate the channel and obtain the distribution of the fading coefficient. Wang et al. [4] propose channel estimation methods for multiuser massive MIMO systems assisted by LIS and present alternatives to decrease the training time necessary to have complete knowledge of the channel coefficients. Tataria et al. [5] discuss practical aspects of real-time implementation of LIS, especially in terms of processing and applications in radio frequency (RF) communications. Elbir et al. [6] present a deep learning framework for channel estimation, considering the massive MIMO scenario using mm-Wave.

Yu et al. [7] propose the use of LIS to improve the coverage of a cellular IoT in the so-called beyond fifth-generation (B5G). The LIS project aims to minimize the energy consumption and study the impact of channel parameters on spectral efficiency.

Ye et al. [8] propose techniques to minimize the symbol error rate (SER) by optimizing the phase shifts and the precoder for a MIMO reconfigurable intelligent surface (RIS) considering a finite alphabet of symbols. Among the strategies proposed are to fix the phase shifts and obtain the optimal precoder or to fix the precoder and find the phase shifts, that solution is useful to reduce the dimensionality of the optimization task and also the performance of the proposed RIS strategy is compared with a relay system and we see the advantage of using the techniques proposed by the authors.

Wu et al. [9] develope a mmWave point-to-point communication system assisted by multiple intelligent subsurfaces with passive reflecting elements, and antenna arrays on the transmitter and receiver. The authors derived the system achievable rate and have found the optimal precoding and power allocation for the LIS phase shift design. He et al. [10] investigate the theoretical limits and Cramér-Rao bounds for the LIS performance in a MIMO 5G system dealing with mmWave and considering the existence of a direct path (NLoS and LoS).

Dardari [11] derives analytical expressions for the channel gain and the spatial degrees-of-freedom (DoF) for the optimal LIS design considering MIMO systems. The analysis is based on electromagnetic theory and employs only geometric arguments. Jung et al. [12] consider that a MIMO system assisted by LIS can be modeled as an LoS after phase cancellation. The authors also analyze the theoretical limitations of the practical system’s performance considering spatially correlated Rician channels and demonstrate that the NLoS component can be neglected when the number of antennas increases.

Yan et al. [13] present a multiuser MIMO (Mu-MIMO) system in which intelligent electromagnetic reflectors perform passive beamforming. The authors also propose to design a receiver with two estimation modules. One for the signal transmitted by the base station and the other, to estimate the additional On/Off information associated with the reflectors that modulate the digital signal arriving at them.

Badiu et al. [14] shows that the perfect estimation of the reflection angles at the LIS array is unfeasible, so we have to model the phase errors due to the estimation and discretization errors. The authors claim that the overall channel, including the LIS, can be modeled as Nakagami-*m* distributed, for phase errors having a generic distribution.

Cavers [15] defines maximal ratio transmission (MRT), establishing that the base station applies a vector of complex weights to compensate the downlink channel by canceling the phase and perform a signal reinforcement. He also shows a generalization for the effects of fading when the system has multiple users, although there is no exact generic solution for the optimal precoder in this scenario.

Makarfi et al. [16] propose to apply reconfigurable intelligent surfaces to expand coverage and improve the signal-to-noise ratio of a vehicular network, which can be seen as a case study of the IoT area using this new massive MIMO solution. The authors explore the idea of using smart radio environments for IoT problems and discuss some relevant aspects beyond 5G to establish communication between vehicles.

Qian et al. [17] present a MIMO system that uses LIS and has an array of antennas on the transmitter and receiver. The signals suffer uncorrelated Rayleigh fading in each channel. The authors obtain good approximations and performance studies based on analytical derivations of the statistical moments associated with the largest eigenvalues of the Wishart matrices related to the LoS and NLoS component. Without losing generality, they assume that the largest eigenvalues have a Gamma distribution and their moments are a function of the number of LIS elements and the number of antennas in the array.

Björnson et al. [18] discuss how the correlation matrix of the LIS elements can be computed under certain conditions, considering Rayleigh fading channels with a direct path between the signal and the final user. In this study, the authors make a more geometric analysis of the problem considering a rectangular panel formed by several reflectors and their constructive parameters, thereby establishing a relationship between the degrees of freedom of the LIS and the rank of the autocorrelation matrix of the reflector panel. Asymptotic analyzes of the SNR variation and channel hardening are also performed when the number of antennas and reflectors increase.

In this work, we investigate the performance of system employing LIS, also known as large reflective surfaces (LRS), taking into account Rayleigh channels and phase errors due to imperfect channel phase cancellation. This work is very general since it considers a direct link between the base station with multiple antennas and the single user. We investigate the system performance and quality of the proposed approximations for channel distribution in terms of the Kullback-Leibler divergence metric. We also present analytical expressions for the bit error probability and a very tight upper bounds for different scenarios in terms of the Von Misses parameter.

Note that the phase estimation errors is modeled as zero mean Von Mises distribution [14], which has a concentration parameter, κ, that helps us model the accuracy of the estimation. Large values of the Von Mises κ implies small errors, when κ→∞ the zero mean Von Mises probability density function is impulsive at zero, and for κ=0 the probability distribution is the uniform distribution.

Analytically obtaining the equivalent fading distribution and the bit error probability is quite intricate due to the considerable sums and transformations of random variables with distinct distributions.

This paper tries to cover some gaps in the literature, with regard to obtaining analytical solutions in the form of simple algebraic expressions involving the channel parameters and the Von Mises parameter, in this way we were able to simplify the analysis of a very generalist system model that may include the direct link with the user, may have one or more antennas at the base station and we maintain the validity of the analysis even when the phase correction algorithm is not efficient. Badiu et al. [14] use the central limit theorem to solve a simpler scenario with one Rayleigh NLoS channel and a rician LoS channel, but considering a single antenna transmitter. Björnson et al. [18] propose asymptotic approaches in a scenario that takes into account the correlation of the LIS elements but is also restricted to the context with a single antenna transmitter which simplifies the analytical solutions.

For organizational purposes, we summarize what is covered in each section. In Section 2, we define the mathematical notation used in the next chapters, in Section 3, the problem is well described mathematically. Section 4 discusses about the effect of phase correction errors. In Section 5, we propose an approximation for the SNR distribution and the error probability. In Section 7, we present the Monte Carlo simulations and the analytical results. Finally, in Section 8, we make our final considerations about the system performance. For easier reading of this paper, the analytical calculations of the mean and variance of the fading coefficient and the Von Mises trigonometric moments are left for the Appendix C.

## 2. Notation

The mathematical notation adopted in this paper considers E|X|, var(X), and cov(X) as the expected value, variance, and covariance of the random variable *X*, respectively. The function $I_{p}\left(\kappa \right)$ is the modified Bessel function of first kind and order *p*, and Q(.) is the Gaussian error function. The operation A∘B is the Haddamard tensor product between the tensors A and B. The term $X^{H}$ represents the Hermitian of the complex matrix X (transposed conjugate) and z∈C is an element of the set of complex numbers.

## 3. System Model

In this section, we describe the mathematical model adopted in this paper and present the rationale to justify the models used for the channel, the distribution of the fading coefficients in the direct (LoS) and indirect (NLoS) links, in addition to the probability distribution associated with the error of phase accomplished by the LIS when performing the beamforming.

**Figure 1:**
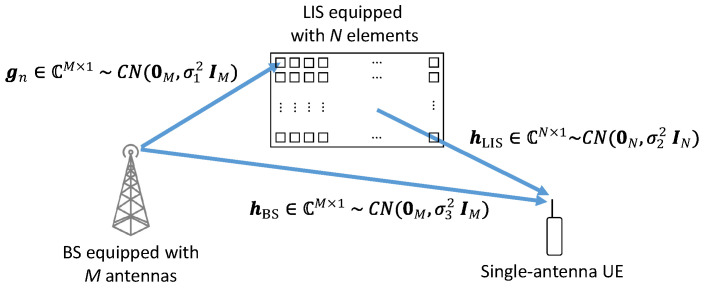
System Model.

This paper considers a multiple-input single-output (MISO) system between a base station (BS) equipped with an antenna array composed of *M* antennas and a single-antenna user as shown in Figure 1. The signal path passes through the LIS environment dividing the system fading in an LoS component between the BS and the user. There are two indirect paths, between each antenna and the LIS reflector, and between each reflector and the user, these indirect links form a composite channel between the base station and the user.We suppose that the LIS is far from the BS, and the user is also far from the LIS. So, the fading coefficients are modeled as uncorrelated Rayleigh.

In this formulation the signal received by the user can be written as

(1) $$y=\left(h_{LIS}^{H}\Phi^{H}G^{H}+h_{BS}^{H}\right)x+\zeta ,$$

where $h_{LIS}\in C^{N\times 1}$ is the Rayleigh channel between the LIS and the user, $G=\left[g_{1}\ldots g_{N}\right]\in C^{M\times N}$ is the Rayleigh channel between the BS and the LIS, $h_{BS}\in C^{M\times 1}$ is the complex normal fading of the direct path between the antenna array and the user (LoS component), $x\in C^{M\times 1}$ is the transmitted symbol after precoding and $\Phi =\mathrm{diag}\left(\left[e^{-j\phi_{1}}\ldots e^{-j\phi_{N}}\right]\right)\in C^{N\times N}$ is a diagonal matrix representing the response of the LIS where $\phi_{n}\in \left[0,2\pi \right],\;\forall n$ is the adjustable phase-shift produced by the *n*th LIS’s element. The variable ζ∼CN(0,1) is the additive white Gaussian noise (AWGN) term. The Tx signal x is defined as x=us where $u\in C^{M\times 1}$ is the precoding vector and s∼CN(0,1) is the data symbol. The precoding vector u is applied by the antenna array at the BS before the transmission.

Considering the MRT criterion, the optimal precoder is given as [19]

(2) $$u=\sqrt{p}\frac{w^{H}}{\left\|w\right\|}$$

where $\sqrt{p}$ is the precoder gain and w is the overall channel defined as

(3) $$w=h_{LIS}^{H}\Phi^{H}G^{H}+h_{BS}^{H}.$$

Let $\eta_{ki}$ and $\theta_{i}$ be the phases of $g_{ki}$ and $h_{i}^{LIS}$, respectively. Therefore, we can rewrite each channel fading coefficient as

(4) $$w_{k}=\sum_{i=1}^{N}\left|g_{ki}\right|\left|h_{i}^{LIS}\right|e^{j\left(\phi_{i}-\theta_{i}-\eta_{ki}\right)}+h_{k}^{BS}$$

From (4), we see that the best situation occurs when the composite fading coefficient is perfectly corrected by the LIS and we can state that $\phi_{i}=\theta_{i}+\eta_{ki}$. But this scenario is unfeasible because perfect channel state information is not a very realistic assumption. Therefore, both cases are approached: (i) the case where the LIS is able to perform perfect phase cancellation, and; (ii) the case where imperfect cancellation is assumed.

Considering the first case, the composite channel can be written as

(5) $$w_{k}=\sum_{i=1}^{N}\left|g_{ki}\right|\left|h_{i}^{LIS}\right|+h_{k}^{BS}.$$

Let $C_{BS,k}=Re\left\{h_{k}^{BS}\right\}$ and $=S_{BS,k}=Im\left\{h_{k}^{BS}\right\}$. Then, the square of the fading vector norm can be written as ${\left\|w\right\|}^{2}=w^{H}w$ and

(6) $${\left\|w\right\|}^{2}=\sum_{k=1}^{M}\left[{\left(\sum_{i=1}^{N}\left|g_{ki}\right|\left|h_{i}^{LIS}\right|+C_{BS,k}\right)}^{2}+S_{BS,k}^{2}\right].$$

To evaluate the system performance and understand the relationship between the bit error rate and the energy per bit applied by the transmitter, we need to know how the fading coefficients of the overall channel are distributed. Therefore, we need to obtain the statistical moments and the distribution of ${\left\|w\right\|}^{2}$.

## 4. Von Mises Distributed Continuous Phase Estimation Errors

Since the phase adjustments performed by the intelligent reflectors are imperfect and cannot completely cancel the channel phase, a term associated with the phase error appears in the equation of the composite channel phase.

Consider that $\phi_{i}=\theta_{k}+\eta_{ki}+\delta_{ki}$ is the phase correction performed by the LIS, so the fading coefficients for each antenna is

(7) $$w_{k}=\sum_{i=1}^{N}\left|g_{ki}\right|\left|h_{i}^{LIS}\right|e^{j\delta_{ki}}+h_{k}^{BS}$$

where the term $\delta_{ki}$ is the phase error, here supposed as Von Mises distributed with probability density function

(8) $$f_{\Delta }\left(\delta \right)=\frac{1}{2\pi I_{0}\left(\kappa \right)}e^{\kappa \cos\delta }.$$

Therefore, we have that

(9) $$w=\left(|G|\circ \Delta \right)\left|h_{LIS}\right|+h_{BS}^{H},$$

This error model considers a matrix $\Delta \in C^{M\times N}$ in which we have the Von Mises phase errors and the Haddamard product is an elementwise product between the phase errors and each channel fading magnitude.

In this case, the moment generating function (MGF) of the Von Mises distribution is useful to obtain the trigonometric moments that are needed to obtain the mean and variance of the fading coefficients. For a random variable δ Von Mises distributed, the MGF can be calculated by

(10) $$E\left[e^{jp\delta }\right]=\alpha_{p}+j\beta_{p},$$

where $\alpha_{p}=\frac{I_{p}\left(\kappa \right)}{I_{0}\left(\kappa \right)}$ and $\beta_{p}=0$ are defined in terms of the modified Bessel function of first kind.

## 5. Approximated Gamma Fading Distribuition

Since each fading coefficient $w_{k}$ is the summation of independent and equally probable random variables, we can apply the central limit theorem (CLT). So, for large values of *N* each $w_{k}$ is approximately complex Gaussian. The term ${\left\|w\right\|}^{2}$ is the sum of squared Gaussian random variables whose generalized distribution is the Gamma distribution. Let V∼Γ(α,β) be a Gamma-distributed variable with shape parameter α and rate parameter β, therefore its probability density function is given as

(11) $$f_{V}\left(v;\alpha ,\beta \right)=\frac{\beta^{\alpha }v^{\alpha -1}e^{-\beta v}}{\Gamma (\alpha )}$$

Note that the mean of the Gamma random variable *V* is given by $E\left[V\right]=\frac{\alpha }{\beta }$, and the variance as $\mathrm{var}\left[V\right]=\frac{\alpha }{\beta^{2}}$[20]. We can compute the mean and variance of ${\left\|w\right\|}^{2}$, denoted here as $\mu_{{\left\|w\right\|}^{2}}$ and $\sigma_{{\left\|w\right\|}^{2}}^{2}$, respectively, and match with E[V] and var[V]. Using this rationale, the following can be written: $\mu_{{\left\|w\right\|}^{2}}=\frac{\alpha }{\beta }$ and $\sigma_{{\left\|w\right\|}^{2}}^{2}=\frac{\alpha }{\beta^{2}}$. Solving this linear equation system, we get that

(12) $$\alpha_{{\left\|w\right\|}^{2}}=\frac{\mu_{{\left\|w\right\|}^{2}}^{2}}{\sigma_{{\left\|w\right\|}^{2}}^{2}},\;\beta_{{\left\|w\right\|}^{2}}=\frac{\mu_{{\left\|w\right\|}^{2}}}{\sigma_{{\left\|w\right\|}^{2}}^{2}}$$

therefore, with the mean and variance of ${\left\|w\right\|}^{2}$, we can generate its Gamma approximated probability density function. Although the idea might seem very simple, the mean and variance of the channel norm are very difficult to be obtained. For the sake of clarity, we detail these calculations in Appendix A, for the case where there are no phase errors. For this case, the mean $\mu_{{\left\|w\right\|}^{2}}$ and variance $\sigma_{{\left\|w\right\|}^{2}}^{2}$, are given in (A17) and (A45), respectively. In the same way, for the case where phase error occurs, Appendix B presents the mean $\mu_{{\left\|w\right\|}^{2}}$ and variance $\sigma_{{\left\|w\right\|}^{2}}^{2}$ as in (A76) and (A91), respectively.

The trigonometric moments needed to perform the calculations are in the Appendix C.

### Kullback–Leibler Divergence

To evaluate the accuracy of approximating ${\left\|w\right\|}^{2}$ as a Gamma random variable, we can use Kullback-Leibler divergence [21]. The Gamma distribution will be compared with the simulation obtained by the Monte Carlo method.

Although the distribution of the fading coefficient is continuous, for purposes of numerical calculation, we estimate the PDF with a finite number of points and thus we also sample the Gamma distribution and calculate the Kullback-Leibler divergence in its discrete form [21]

(13) $$D_{KL}\left(D_{1}\left|\right|D_{2}\right)=\sum_{x\in \chi }d_{1}\left(x\right)\;\log\left(\frac{d_{1}\left(x\right)}{d_{2}\left(x\right)}\right),$$

where $D_{1}$ and $D_{2}$ are the simulated and the theoretical distributions, respectively, whose probability distribution functions are $d_{1}\left(x\right)$ and $d_{2}\left(x\right)$ respectively and χ is the set of points available to represent the distributions.

## 6. Error Probability Calculations

The error probability for the *M*-QAM modulation can be approximately obtained by [22]

(14) $$P_{e}^{QAM}\left(\gamma \right)=1-{\left(1-2\left(1-\frac{1}{\sqrt{M}}\right)Q\left[\sqrt{\frac{3\gamma \log_{2}M}{\left(M-1\right)}}\right]\right)}^{2},$$

where M is the size of the *M*-QAM constellation. Under Gamma fading, the mean error probability can be calculated as

(15) $${\bar{P}}_{e}^{QAM}\left(\gamma \right)=\int_{0}^{\infty }P_{e}^{QAM}\left(\gamma v\right)f_{{\left\|w\right\|}^{2}}\left(v\right)dv,$$

where $\gamma =p\gamma_{0}$ and $\gamma_{0}$ is the SNR at the receiver while ${\bar{P}}_{e}^{QAM}$ is the mean error probability considering the fading coefficient *v* and the Gamma pdf $f_{{\left\|w\right\|}^{2}}\left(v\right)$.

Therefore the error probability can be expressed as

(16) $${\bar{P}}_{e}^{QAM}\left(\gamma \right)=\int_{0}^{\infty }P_{e}^{QAM}\left(\gamma v\right)\frac{\beta^{\alpha }v^{\alpha -1}e^{-\beta v}}{\Gamma (\alpha )}dv,$$

where $\alpha =\alpha_{{\left\|w\right\|}^{2}}$ and $\beta =\beta_{{\left\|w\right\|}^{2}}$ are calculated by (12).

In [23], we have a useful approximation for the bit error probability on an M-QAM schema. Considering coherent detection, we can state that

(17) $$P_{e}^{QAM}\left(\gamma \right)\approx \frac{4}{\log_{2}M}Q\left(\sqrt{\frac{3\gamma \log_{2}M}{M-1}}\right)$$

By using the approximation in (17), we propose an upper bound for the bit error probability for the transmission of *M*-QAM symbols under Gamma fading by applying the Chernoff bound $Q\left(x\right)<-\frac{1}{2}e^{-\frac{1}{2}x^{2}}$ and solving the integral formula in (16). The proposed bound for error probability can be calculated as

(18) $${\bar{P}}_{e}^{QAM}\left(\gamma \right)<\frac{1.38629{\left(\frac{2.16404\gamma \log(M)}{\left(M-1\right)\beta_{{\left\|w\right\|}^{2}}}+1\right)}^{-\alpha_{{\left\|w\right\|}^{2}}}}{\log(M)}$$

**Figure 2:**
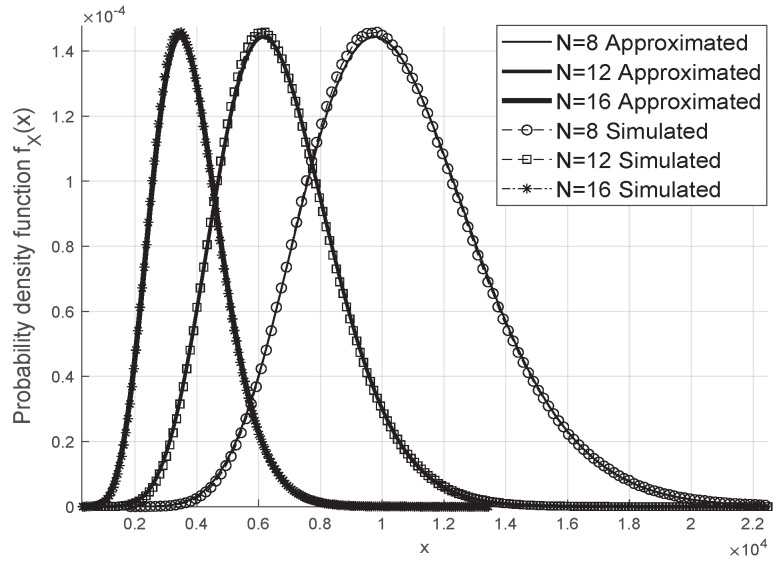
Approximated Gamma Distribution of ${\left\|w\right\|}^{2}$ without phase errors.

**Figure 3:**
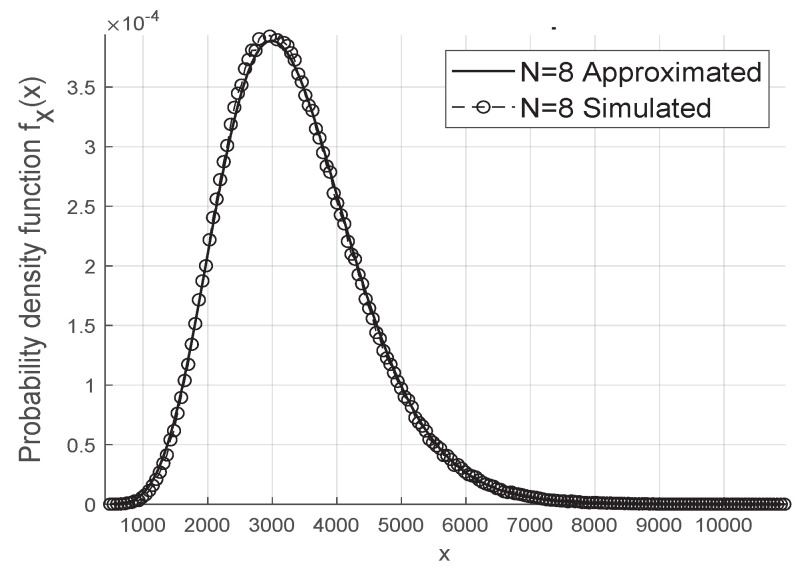
Approximated Gamma Distribution of ${\left\|w\right\|}^{2}$ with Von Mises κ=2 errors.

The gamma approximation for the resulting fading coefficient is adequate and works even for small values of *N* and *M* when we consider the scenario without phase errors, as in Figure 2, and in the case where we have the Von Mises distributed phase errors as shown in Figure 3.

**Figure 4:**
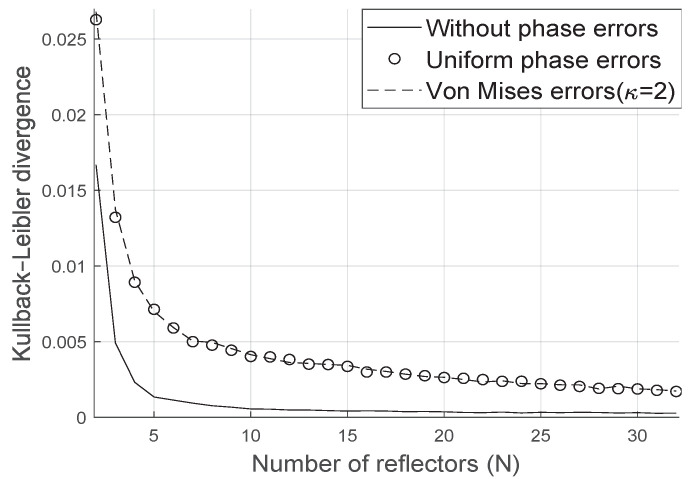
Kullback–Leibler divergence for the fading squared magnitude.

As we can see in Figure 4 the Kullback-Leibler divergence decays with the number of reflectors at the LIS and the distribution is well represented by the proposed gamma approximation.

## 7. Simulated Results

We have simulated the bit error probability and generated the fading coefficients of all LoS and NLoS channels using the Monte Carlo method with $10^{6}$ iterations. We have assumed that the phase errors follow the uniform or the Von Mises distribution. To calculate the error probability, we have solved numerically the integral in (16) and compared it with the Monte Carlo simulation.

**Figure 5:**
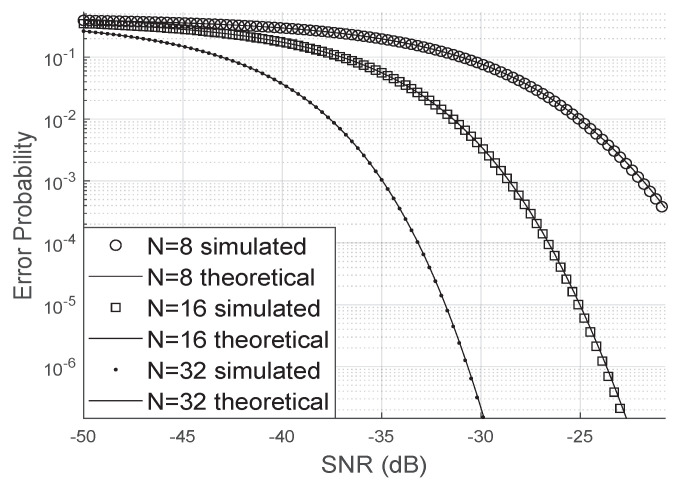
Error probability without phase errors.

Figure 5 shows the simulated bit error rate when there are no phase errors. As it can be observed, the simulation is very close to the analytical bit error probability. Moreover, as the number of LIS reflectors increases, the bit error probability decreases faster concerning the SNR. This result is valid even for small values of *N*, for example, for N=8.

**Figure 6:**
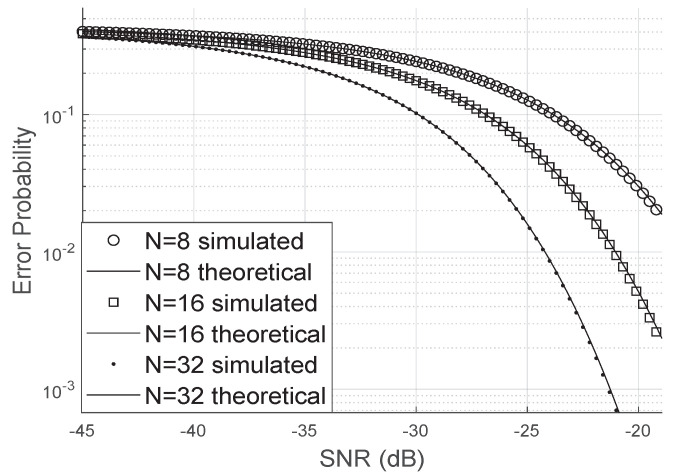
Error probability for uniformly distributed phase errors.

On the other hand, when the phase error is uniformly distributed, as shown in Figure 6, the bit error probability increases significantly compared to the scenario where there are no phase errors. Again, we can observe that the analytical curve matches perfectly the simulated results, even for a small number of antennas or a small number of elements at the LIS.

It is worth mentioning that a uniform phase error, as pointed out by [24], may mean that the LIS’s channel estimation or phase correction was not so effective since large and small phase errors are equiprobable.

**Figure 7:**
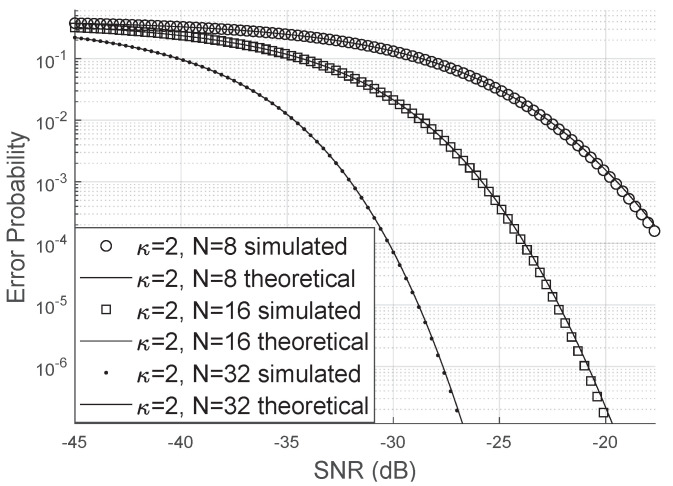
Error probability for Von Mises distributed phase errors.

Comparing Figure 6 and Figure 7, we can see that the error probability is smaller when κ>0. The occurrence of small errors is more probable than large errors (±π).

**Figure 8:**
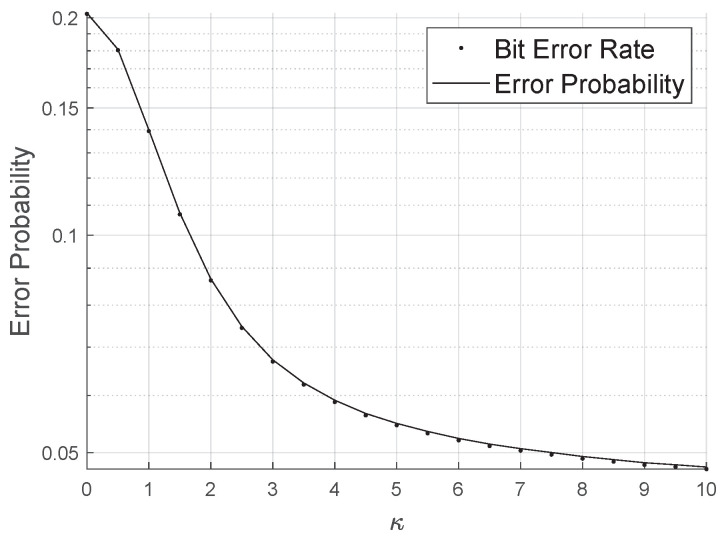
Error probability varying the Von Mises concentration parameter.

Figure 8 shows how the bit error probability behaves as the concentration parameter varies for SNR of −25 dB. It is clear that the rate decreases as κ increases. Therefore, the κ parameter of the LIS can be considered a qualitative parameter of the phase correction performed by the reflectors for a specific channel estimation method.

**Figure 9:**
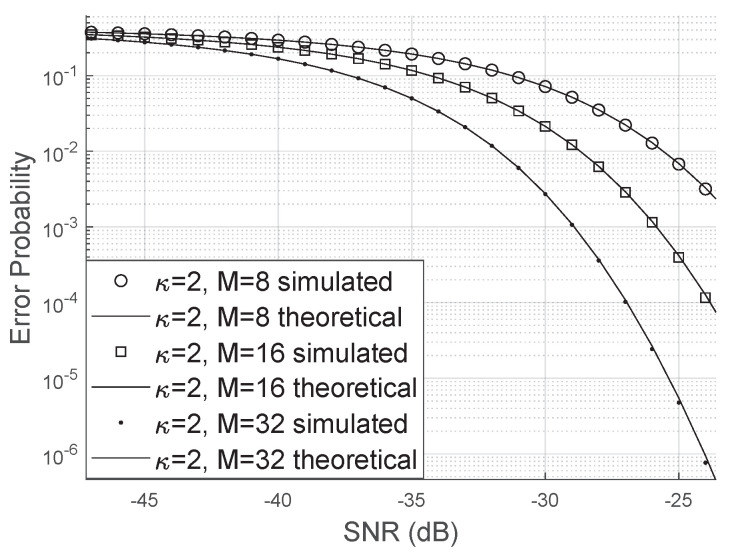
Error probability varying the size of the antenna array.

In Figure 9, we vary the size of the antenna array at BS and note that the bit error rate decreases significantly when *M* increases. Furthermore, our approximation is valid for both large and small values of *M*.

**Figure 10:**
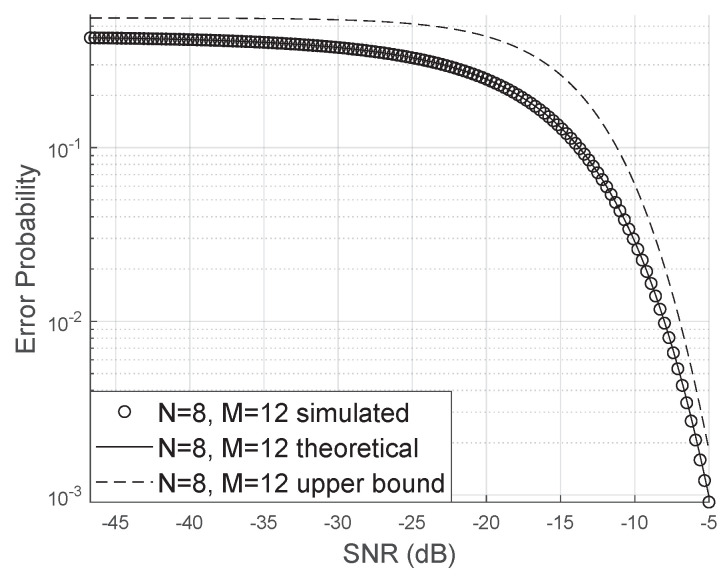
Proposed upper bound for the error probability.

Although the numerical calculation of the analytical expression of the bit error probability is computationally fast, it may still be interesting to use a direct expression that does not involve solving numerical integrals. The proposed upper bound is very close to the simulated results, as we can see in Figure 10.

**Figure 11:**
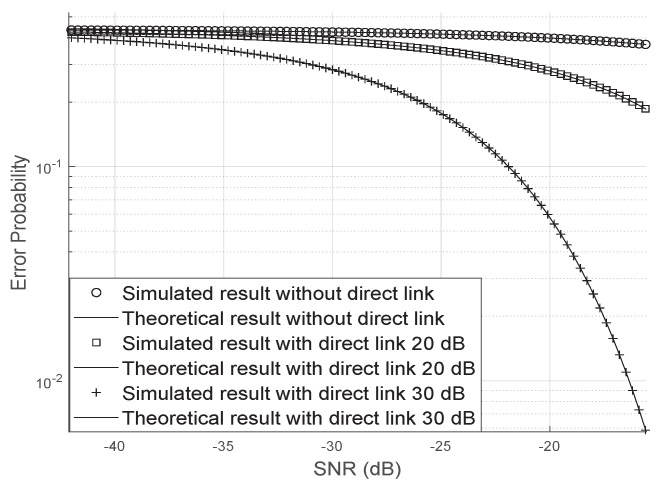
Effect of the direct link in the bit error probability.

Figure 11 shows the influence of the direct link in the bit error probability. In this figure, we have assumed that the direct link is 10 dB and 30 dB larger than the two indirect links. As it can be seen, when the direct link is strong, the error probability decays quickly with the increase of the SNR, on the other hand, when the direct link is weak, the bit error probability requires a larger SNR to decrease.

## 8. Final Considerations

This paper has presented how some parameters like number of antennas at BS and electromagnetic reflectors at LIS, channel, and phase error distribution can influence on the performance of a massive MIMO system assisted by LIS.

Assuming that there is the direct link between the user and the base station and phase errors performed by the LIS, we have derived analytical expressions for the channel probability distribution function and bit error probability. As conclusion, all the sum of the fading coefficients and phase noise involved in the MIMO communication system assisted by LIS can be modeled as a Gamma random variable.

We have proved the accuracy of our approximation through the Kullback-Leibler divergence even when the phase error follows either the uniform or the Von Mises distribution with arbitrary concentration parameter. In the absence of phase error, the divergence between the simulated distribution and the proposed analytical approach decreases even faster with the increase of the number of reflectors at the LIS.

Further studies may include expanding this analysis to more general fading distributions such as Nakagami-*m*, which include the Rayleigh distribution as special case and can reasonably approximate Rician fading channels for large values of *m*. 

## Appendix A

### Appendix A.1. Mean of $w_{k}$ Given in (5)

Departing from (5), the mean of the each total fading coefficient can be calculated as

(A1) $$E\left[w_{k}\right]=E\left[\sum_{i=1}^{N}\left|g_{ki}\right|\left|h_{i}^{LIS}\right|+h_{k}^{BS}\right]$$

The mean of the product of two random variables *X* and *Y* is given by $\mathrm{var}\left(XY\right)=\mathrm{var}\left(X\right)\mathrm{var}\left(Y\right)+\mathrm{var}\left(X\right)E{\left[Y\right]}^{2}+\mathrm{var}\left(X\right)E{\left[Y\right]}^{2}$ [20], since $|g_{ki}|$ and $|h_{k}^{LIS}|$ are independent and equally probable on the summation variable. So,

(A2) $$E\left[w_{k}\right]=N\times E\left[\left|g_{ki}\right|\right]E\left[\left|h_{i}^{LIS}\right|\right]+E\left[h_{k}^{BS}\right]$$

Since $E\left[h_{k}^{BS}\right]=0$,

(A3) $$E\left[w_{k}\right]=N\times E\left[\left|g_{ki}\right|\right]E\left[\left|h_{i}^{LIS}\right|\right]$$

The terms $|g_{ki}|$ and $\left|h_{i}^{LIS}\right|$ are Rayleigh distributed, since the variables $g_{ki}$ and $h_{i}^{LIS}$ are zero mean with variances $\sigma_{1}^{2}$ and $\sigma_{2}^{2}$ respectively. Therefore

(A4) $$E\left[\left|g_{ki}\right|\right]=\sigma_{1}\sqrt{\frac{\pi }{2}},\;E\left[\left|h_{i}^{LIS}\right|\right]=\sigma_{2}\sqrt{\frac{\pi }{2}}.$$

Let $w_{k}=c_{k}+js_{k}$, where $c_{k}$ and $s_{k}$ are the in-phase and quadrature components of the fading coefficient with respect to the antenna *k*. Also, let $\mu_{c_{k}}=E\left[c_{k}\right]$ and $\mu_{s_{k}}=E\left[s_{k}\right]=0$. Then,

(A5) $$E\left[w_{k}\right]=\mu_{c_{k}}=N\frac{\pi }{2}\sigma_{1}\sigma_{2}.$$

### Appendix A.2. Variance of $w_{k}$ Given in (5)

The variance of $w_{k}$ is given by

(A6) $$\begin{array}{c} \mathrm{var}\left(w_{k}\right)=E\left[\left(c_{k}+js_{k}\right)\left(c_{k}-js_{k}\right)\right]-{\left(E\left[(c_{k}+js_{k})\right]\right)}^{2}=\mathrm{var}\left(c_{k}\right)+\mathrm{var}\left(s_{k}\right), \end{array}$$

where

(A7) $$\mathrm{var}\left(c_{k}\right)=\mathrm{var}\left(\sum_{i=1}^{N}\left|g_{ki}\right|\left|h_{i}^{LIS}\right|+C_{BS,k}\right),$$

and can be expanded as

(A8) $$\mathrm{var}\left(c_{k}\right)=\mathrm{var}\left(\sum_{i=1}^{N}\left|g_{ki}\right|\left|h_{i}^{LIS}\right|\right)+\mathrm{var}\left(C_{BS,k}\right)+2{\left(E\left[C_{BS,k}\sum_{i=1}^{N}\left|g_{ki}\right|\left|h_{i}^{LIS}\right|\right]\right)}^{2},$$

The first term of (A8) can be written as

(A9) $$\mathrm{var}\left(\sum_{i=1}^{N}\left|g_{ki}\right|\left|h_{i}^{LIS}\right|\right)=N\mathrm{var}\left(|g_{ki}|\left|h_{i}^{LIS}\right|\right)$$

Note in (A9) that it is necessary to compute the variance of the product of two random variables. Let *X* and *Y*, two independent random variables, then $\mathrm{var}\left(XY\right)=\mathrm{var}\left(X\right)\mathrm{var}\left(Y\right)+\mathrm{var}\left(X\right)E{\left[Y\right]}^{2}+\mathrm{var}\left(Y\right)E{\left[X\right]}^{2}$ and then.

(A10) $$\mathrm{var}\left(|g_{ki}|\left|h_{i}^{LIS}\right|\right)=\mathrm{var}\left(|g_{ki}|\right)\times \mathrm{var}\left(\left|h_{i}^{LIS}\right|\right)\;+\mathrm{var}\left(|g_{ki}|\right){\left(E\left[\left|h_{i}^{LIS}\right|\right]\right)}^{2}+\mathrm{var}\left(\left|h_{i}^{LIS}\right|\right){\left(E\left[|g_{ki}|\right]\right)}^{2}$$

Since $\left|g_{ki}\right|$ and $\left|h_{i}^{LIS}\right|$ are Rayleigh distributed,

(A11) $$\mathrm{var}\left(\left|g_{ki}\right|\right)=\frac{4-\pi }{2}\sigma_{1}^{2},\;\;\mathrm{var}\left(\left|h_{i}^{LIS}\right|\right)=\frac{4-\pi }{2}\sigma_{2}^{2}$$

and so, after some simplifications, we have that

(A12) $$\mathrm{var}\left(|g_{ki}|\left|h_{i}^{LIS}\right|\right)=\frac{16-\pi^{2}}{4}{\left(\sigma_{1}\sigma_{2}\right)}^{2}$$

Since $E\left[C_{BS,k}\right]=0$ and the terms $C_{BS,k}$ and $\sum_{i=1}^{N}\left|g_{ki}\right|\left|h_{i}^{LIS}\right|$ are independent, then

(A13) $$\mathrm{var}\left(c_{k}\right)=N\frac{16-\pi^{2}}{4}{\left(\sigma_{1}\sigma_{2}\right)}^{2}+\sigma_{3}^{2}$$

therefore the variance of $w_{k}$ is given as

(A14) $$\mathrm{var}\left(w_{k}\right)=N\frac{16-\pi^{2}}{4}{\left(\sigma_{1}\sigma_{2}\right)}^{2}+2\sigma_{3}^{2}.$$

### Appendix A.3. Expected Value of ${\left\|w\right\|}^{2}$

The expected value of ${\left\|w\right\|}^{2}$ can be calculated by

(A15) $$E\left[{\left\|w\right\|}^{2}\right]=E\left[\sum_{k=1}^{M}{\left|w_{k}\right|}^{2}\right]=ME\left[|w_{k}{|}^{2}\right]$$

With $E[w_{k}]$ and $\mathrm{var}(w_{k})$, we can calculate $E\left[|w_{k}{|}^{2}\right]$ as $E\left[|w_{k}{|}^{2}\right]=\mathrm{var}\left(w_{k}\right)+{\left(E\left[w_{k}\right]\right)}^{2}$. Therefore, we have

(A16) $$E\left[|w_{k}{|}^{2}\right]=N\frac{16-\pi^{2}}{4}{\left(\sigma_{1}\sigma_{2}\right)}^{2}+2\sigma_{3}^{2}+{\left(N\frac{\pi }{2}\sigma_{1}\sigma_{2}\right)}^{2},$$

so the mean of the overall channel fading coefficient is

(A17) $$\mu_{{\left\|w\right\|}^{2}}=E\left[{\left\|w\right\|}^{2}\right]=M\left(N\frac{16-\pi^{2}}{4}{\left(\sigma_{1}\sigma_{2}\right)}^{2}+2\sigma_{3}^{2}+{\left(N\frac{\pi }{2}\sigma_{1}\sigma_{2}\right)}^{2}\right)$$

### Appendix A.4. Correlation between the Fading Coefficients

Since the real and imaginary parts of the fading coefficients, without phase errors, are uncorrelated, we analyze only the correlation between the real parts as follows.

(A18) $$\rho_{c_{i},c_{k}}=\frac{E\left[c_{i}c_{k}\right]-E\left[c_{i}\right]E\left[c_{k}\right]}{\sqrt{\mathrm{var}\left(c_{i}\right)\mathrm{var}\left(c_{k}\right)}}.$$

Since $\mathrm{var}\left(c_{i}\right)=\mathrm{var}\left(c_{k}\right)$ and $E\left[c_{i}\right]=E\left[c_{k}\right]$,

(A19) $$\rho_{c_{i},c_{k}}=\frac{E\left[c_{i}c_{k}\right]-\mu_{c_{k}}^{2}}{\mathrm{var}(c_{k})}$$

where

(A20) $$E\left[c_{i}c_{k}\right]=E\left[\left(\sum_{l=1}^{N}|g_{il}\left|\right|h_{l}^{LIS}|+C_{BS,i}\right)\times \right.\left.\left(\sum_{m=1}^{N}|g_{km}\left|\right|h_{m}^{LIS}|+C_{BS,k}\right)\right],$$

and simplifies to

(A21) $$\begin{array}{c} E\left[c_{i}c_{k}\right]=E\left[\sum_{l=1}^{N}\sum_{m=1}^{N}|g_{il}\left|\right|g_{km}\left|\right|h_{l}^{LIS}\left|\right|h_{m}^{LIS}|\right]+E\left[C_{BS,i}C_{BS,k}\right]+E\left[C_{BS,i}\right]\left(\sum_{m=1}^{N}|g_{km}\left|\right|h_{k}^{LIS}|+C_{BS,k}\right)+\\ E\left[C_{BS,k}\right]\left(\sum_{l=1}^{N}\left|g_{il}\right|\left|h_{i}^{LIS}\right|+C_{BS,i}\right). \end{array}$$

Since $E\left[C_{BS,i}\right]=E\left[C_{BS,k}\right]=0$, $E\left[|g_{il}|\right]=E\left[|g_{km}|\right]$, $E\left[|h_{l}^{LIS}|\right]=E\left[|h_{m}^{LIS}|\right]$ so

(A22) $$E\left[c_{i}c_{k}\right]=\sum_{l=1}^{N}\sum_{m=1}^{N}E\left[|g_{il}\left|\right|g_{km}\left|\right|h_{l}^{LIS}\left|\right|h_{m}^{LIS}|\right]+E\left[C_{BS,i}C_{BS,k}\right]$$

Since $|g_{il}|$ and $|g_{km}|$ are independent, therefore

(A23) $$E\left[|g_{il}\left|\right|g_{km}\left|\right|h_{l}^{LIS}\left|\right|h_{m}^{LIS}|\right]=E\left[|g_{il}|\right]E\left[|g_{km}|\right]E\left[|h_{l}^{LIS}|\right]E\left[|h_{m}^{LIS}|\right]\;\forall l\neq m$$

and

(A24) $$l=m\Rightarrow E\left[\left|g_{il}\right|\left|g_{km}\right|\left|h_{l}^{LIS}\right|\left|h_{m}^{LIS}\right|\right]=E\left[|g_{il}|\right]E\left[|g_{km}|\right]E\left[|h_{m}^{LIS}{|}^{2}\right]$$

since

(A25) $$E\left[{\left|h_{m}^{LIS}\right|}^{2}\right]=\mathrm{var}\left(|h_{m}^{LIS}|\right)+{\left(E\left[|h_{m}^{LIS}|\right]\right)}^{2},$$

so we have that

(A26) $$E\left[{\left|h_{m}^{LIS}\right|}^{2}\right]=\frac{4-\pi }{2}\sigma_{2}^{2}+\frac{\pi }{2}\sigma_{2}^{2}=2\sigma_{2}^{2}$$

Therefore

(A27) $$\sum_{l=1}^{N}\sum_{m=1}^{N}E\left[|g_{il}\left|\right|g_{km}\left|\right|h_{l}^{LIS}\left|\right|h_{m}^{LIS}|\right]=N\pi \sigma_{1}^{2}\sigma_{2}^{2}\left(1+\frac{(N-1)\pi }{4}\right)$$

Since

$$E\left[C_{BS,i}C_{BS,k}\right]=\left\{\begin{array}{cc} 0 & k\neq \;i\\ E\left[C_{BS,k}^{2}\right]=\sigma_{3}^{2} & k=i \end{array}\right.$$

Therefore

$$E\left[c_{i}c_{k}\right]=\left\{\begin{array}{cc} N\pi \sigma_{1}^{2}\sigma_{2}^{2}\left(1+\frac{(N-1)\pi }{4}\right) & k\neq \;i\\ N\pi \sigma_{1}^{2}\sigma_{2}^{2}\left(1+\frac{(N-1)\pi }{4}\right)+\sigma_{3}^{2} & k=i \end{array}\right.$$

Since $E\left[c_{k}\right]=E\left[w_{k}\right]=N\frac{\pi }{2}\sigma_{1}\sigma_{2}$, we only need to consider the case i≠k, because for i=k we have that $\rho_{c_{i},c_{k}}=\rho_{c_{k},c_{k}}=1$ therefore

(A28) $$\rho_{c_{i},c_{k}}=\frac{N\pi \sigma_{1}^{2}\sigma_{2}^{2}\left(1+\frac{(N-1)\pi }{4}\right)-{\left(\frac{N\pi }{2}\sigma_{1}\sigma_{2}\right)}^{2}}{N\frac{16-\pi^{2}}{4}{\left(\sigma_{1}\sigma_{2}\right)}^{2}+\sigma_{3}^{2}}\forall i\neq k$$

by performing algebraic simplifications we have that

(A29) $$\rho_{c_{i},c_{k}}=\frac{N\pi {\left(\sigma_{1}\sigma_{2}\right)}^{2}}{N\left(\pi +4\right){\left(\sigma_{1}\sigma_{2}\right)}^{2}+\frac{4}{4-\pi }\sigma_{3}^{2}}\forall i\neq k$$

### Appendix A.5. Variance of ${\left\|w\right\|}^{2}$

Let $Z_{k}={\left|w_{k}\right|}^{2}$, so the variance of the sum of the correlated random variables $Z_{k}$ will be given by

(A30) $$\mathrm{var}\left({\left\|w\right\|}^{2}\right)=\mathrm{var}\left(\sum_{i=1}^{M}Z_{i}\right)=\sum_{i=1}^{M}\mathrm{var}\left(Z_{i}\right)+\;2\sum_{1\leq i<k\leq M}\mathrm{cov}\left(Z_{i},Z_{k}\right).$$

Since the variables have the same distribution and parameters therefore

(A31) $$\mathrm{var}\left(\sum_{i=1}^{M}Z_{i}\right)=M\times \mathrm{var}\left(Z_{i}\right)+M\left(M-1\right)\times \mathrm{cov}\left(Z_{i},Z_{k}\right),$$

so the pairwise covariance can be obtained by

(A32) $$\mathrm{cov}\left(Z_{i},Z_{k}\right)=E\left[Z_{i}Z_{k}\right]-E\left[Z_{i}\right]E\left[Z_{k}\right]$$

where

(A33) $$E\left[Z_{i}Z_{k}\right]=E[|w_{i}{|}^{2}|w_{k}{|}^{2}]=E\left[\left(c_{i}^{2}+s_{i}^{2}\right)\left(c_{k}^{2}+s_{k}^{2}\right)\right]$$

and simplifies to

(A34) $$E\left[Z_{i}Z_{k}\right]=E\left[c_{i}^{2}c_{k}^{2}\right]+E\left[c_{i}^{2}s_{k}^{2}\right]+E\left[s_{i}^{2}c_{k}^{2}\right]+E\left[s_{i}^{2}s_{k}^{2}\right]$$

Since

(A35) $$E\left[c_{i}^{2}s_{k}^{2}\right]=E\left[s_{i}^{2}c_{k}^{2}\right],$$

so,

(A36) $$E\left[Z_{i}Z_{k}\right]=E\left[c_{i}^{2}c_{k}^{2}\right]+2E\left[c_{i}^{2}s_{k}^{2}\right]+E\left[s_{i}^{2}s_{k}^{2}\right]$$

Considering that $c_{k}$ and $c_{i}$ are correlated Gaussian random variables with the same mean and variance, therefore we have that

(A37) $$E\left[c_{i}^{2}c_{k}^{2}\right]=\int_{-\infty }^{\infty }\int_{-\infty }^{\infty }x^{2}y^{2}f_{c_{i},c_{k}}\left(x,y\right)dxdy$$

where $f_{c_{i},c_{k}}\left(x,y\right)$ is the joint probability density function of the correlated Gaussian random variables $c_{i}$ and $c_{k}$.

(A38) $$E\left[c_{i}^{2}c_{k}^{2}\right]=\mu_{c_{k}}^{4}+2\mu_{c_{k}}^{2}\left(1+2\rho_{c_{i},c_{k}}\right)\sigma_{c_{k}}^{2}+\left(1+2\rho_{c_{i},c_{k}}^{2}\right)\sigma_{c_{k}}^{4}$$

$$E\left[c_{i}^{2}c_{k}^{2}\right]=\left\{\begin{array}{cc} k_{1}^{4}\sigma_{12}^{4}+2k_{1}^{2}\sigma_{12}^{2}\left(\frac{k_{1}}{2\pi }a_{1}a_{2}\sigma_{12}^{2}+\sigma_{3}^{2}\right)+2k_{1}^{3}a_{1}\sigma_{12}^{4}+\frac{1}{2}k_{1}^{2}\sigma_{12}^{4}a_{1}^{2}+{\left(\frac{k_{1}}{2\pi }a_{1}a_{2}\sigma_{12}^{2}+\sigma_{3}^{2}\right)}^{2} & i\neq k\\ k_{1}^{4}\sigma_{12}^{4}+6k_{1}^{2}\sigma_{12}^{2}\left(\frac{k_{1}}{2\pi }a_{1}a_{2}\sigma_{12}^{2}+\sigma_{3}^{2}\right)+3{\left(\frac{k_{1}}{2\pi }a_{1}a_{2}\sigma_{12}^{2}+\sigma_{3}^{2}\right)}^{2} & i=k \end{array}\right.$$

Therefore $E[c_{i}^{2}c_{k}^{2}]$ can be calculated by (A68), where $k_{1}=N\frac{\pi }{2}$, $a_{1}=4-\pi$, $a_{2}=4+\pi$ and $\sigma_{12}=\sigma_{1}\sigma_{2}$.

Since $c_{i}$ and $s_{k}$ are independent and $E[|s_{k}^{2}\left|\right]=\mathrm{var}\left(s_{k}\right)$ so we have that

(A39) $$E\left[c_{i}^{2}s_{k}^{2}\right]=E\left[c_{i}^{2}\right]E\left[s_{k}^{2}\right]$$

Since

$$E\left[c_{i}^{2}\right]=var\left(c_{i}\right)+{\left(E[c_{i}]\right)}^{2}=\frac{k_{1}}{2\pi }a_{1}a_{2}\sigma_{12}^{2}+\sigma_{3}^{2}+k_{1}^{2}\sigma_{12}^{2},$$

so we have that

$$E\left[c_{i}^{2}\right]=\sigma_{12}^{2}\left(\frac{k_{1}}{2\pi }a_{1}a_{2}+k_{1}^{2}\right)+\sigma_{3}^{2},$$

Since $E\left[s_{k}^{2}\right]=var\left(s_{k}\right)=\sigma_{3}^{2}$, therefore

(A40) $$E\left[c_{i}^{2}s_{k}^{2}\right]=\sigma_{123}^{2}\left(\frac{k_{1}}{2\pi }a_{1}a_{2}+k_{1}^{2}\right)+\sigma_{3}^{4}$$

where $\sigma_{123}^{2}=\sigma_{1}^{2}\sigma_{2}^{2}\sigma_{3}^{2}$.

The fourth order moment $E[s_{k}^{4}]$ of a Gaussian random variable is well known in the literature and can be calculated by

(A41) $$E\left[s_{k}^{4}\right]={\left(E\left[s_{k}\right]\right)}^{4}+2{\left(E\left[s_{k}\right]\right)}^{2}\mathrm{var}\left(s_{k}\right)+3{\left(\mathrm{var}\left(s_{k}\right)\right)}^{2}$$

Since $E[s_{k}]=0$, therefore

(A42) $$E\left[s_{k}^{4}\right]=3\sigma_{3}^{4}$$

(A43) $$\begin{array}{c} E[Z_{i}Z_{k}]=\\ \left\{\begin{array}{cc} k_{1}^{4}\sigma_{12}^{4}+2k_{1}^{2}\sigma_{12}^{2}\left(\frac{k_{1}}{2\pi }a_{1}a_{2}\sigma_{12}^{2}+\sigma_{3}^{2}\right)+2k_{1}^{3}a_{1}\sigma_{12}^{4}+\frac{1}{2}k_{1}^{2}\sigma_{12}^{4}a_{1}^{2}+{\left(\frac{k_{1}}{2\pi }a_{1}a_{2}\sigma_{12}^{2}+\sigma_{3}^{2}\right)}^{2}+\ldots \\ +2\sigma_{123}^{2}\left(\frac{k_{1}}{2\pi }a_{1}a_{2}+k_{1}^{2}\right)+3\sigma_{3}^{4} & i\neq k\\ k_{1}^{4}\sigma_{12}^{4}+6k_{1}^{2}\sigma_{12}^{2}\left(\frac{k_{1}}{2\pi }a_{1}a_{2}\sigma_{12}^{2}+\sigma_{3}^{2}\right)+3{\left(\frac{k_{1}}{2\pi }a_{1}a_{2}\sigma_{12}^{2}+\sigma_{3}^{2}\right)}^{2}+2\sigma_{123}^{2}\left(\frac{k_{1}}{2\pi }a_{1}a_{2}+k_{1}^{2}\right)+5\sigma_{3}^{4} & i=k \end{array}\right. \end{array}$$

With the expected value $E[Z_{i}Z_{k}]$, the expected value of $Z_{k}={\left|w_{k}\right|}^{2}$ and the consideration that $E\left[Z_{i}\right]=E\left[Z_{k}\right]\;\forall i\forall k$, the covariance between $Z_{i}$ and $Z_{k}$ can be obtained by

(A44) $$\mathrm{cov}\left(Z_{i},Z_{k}\right)=E\left[Z_{i}Z_{k}\right]-{\left(E[Z_{k}]\right)}^{2}$$

Note that $\mathrm{var}\left(Z_{k}\right)=\mathrm{cov}\left(Z_{k},Z_{k}\right)$, so the variance of ${\left\|w\right\|}^{2}$ can be obtained by

(A45) $$\sigma_{{\left\|w\right\|}^{2}}^{2}=\mathrm{var}\left({\left\|w\right\|}^{2}\right)=M\mathrm{cov}\left(Z_{k},Z_{k}\right)+M\left(M-1\right)\mathrm{cov}\left(Z_{i},Z_{k}\right)$$

By substituting the terms $E[Z_{i}Z_{k}]$ and $E[Z_{k}]$, given in (A43) and (A5), in the Equation (A45), we have finally have the analytical expression of the variance in (A46).

(A46) $$\begin{array}{c} \sigma_{{\left\|w\right\|}^{2}}^{2}=\\ M\left(k_{2}^{4}+2k_{2}^{2}\left(k_{3}+\sigma_{3}^{2}\right)+2k_{1}k_{4}^{2}a_{1}\frac{1}{2}k_{4}^{2}a_{1}^{2}+{\left(k_{3}+\sigma_{3}^{2}\right)}^{2}+2\sigma_{3}^{2}\left(k_{3}+k_{2}^{2}\right)+3\sigma_{3}^{4}-{\left(k_{3}+2\sigma_{3}^{2}+k_{2}^{2}\right)}^{2}\right)+\ldots \\ M\left(M+1\right)\left(k_{2}^{4}+6k_{2}^{2}\left(k_{3}+\sigma_{3}^{2}\right)+3{\left(k_{3}+\sigma_{3}^{2}\right)}^{2}+2\left(k_{3}+k_{2}^{2}\right)+5\sigma_{3}^{4}-{\left(k_{3}+2\sigma_{3}^{2}+k_{2}^{2}\right)}^{2}\right) \end{array}$$

where

$$\begin{array}{c} k_{2}=k_{1}\sigma_{12},\;k_{3}=\frac{k_{1}}{2\pi }a_{1}a_{2}\sigma_{12}^{2},\;k_{4}=k_{1}\sigma_{12}^{2}\; \end{array}$$

## Appendix B. Mean and Variance of the Overall Channel Fading Coefficient with Von Mises Distributed Phase Errors

### Appendix B.1. Mean of ${\tilde{w}}_{k}$

Let ${\tilde{w}}_{k}={\tilde{c}}_{k}+j{\tilde{s}}_{k}$ be the fading coefficient with respect to the antenna *k* when phase errors occurs at the LIS.

To obtain the mean of ${\tilde{w}}_{k}$ we need to calculate the mean of ${\tilde{c}}_{k}$ and ${\tilde{s}}_{k}$.

The mean value of the in-phase fading component ${\tilde{c}}_{k}$ is

(A47) $$E\left[{\tilde{c}}_{k}\right]=N\frac{\pi }{2}\sigma_{12}\alpha_{1}$$

and the quadrature component mean $E[{\tilde{s}}_{k}]$ is

(A48) $$E\left[{\tilde{s}}_{k}\right]=E\left[\sum_{i=1}^{N}\left|g_{ki}\right|\left|h_{i}^{LIS}\right|\sin\delta_{ki}+{\tilde{S}}_{k}^{BS}\right]$$

using the linearity of the expected value we can rewrite the equation as

(A49) $$E\left[{\tilde{s}}_{k}\right]=\sum_{i=1}^{N}E\left[|g_{ki}|\left|h_{i}^{LIS}\right|\sin\delta_{ki}\right]+E\left[{\tilde{S}}_{k}^{BS}\right]$$

since $\beta_{1}=0$, so

(A50) $$E\left[{\tilde{s}}_{k}\right]=N\frac{\pi }{2}\sigma_{12}\beta_{1}=0$$

Therefore we can calculate the mean of the overall fading coefficient, for the antenna *k* by using

(A51) $$E\left[{\tilde{w}}_{k}\right]=E\left[{\tilde{c}}_{k}\right]+E\left[{\tilde{s}}_{k}\right]=N\frac{\pi }{2}\sigma_{12}\alpha_{1}.$$

### Appendix B.2. Variance of the In-Phase and Quadrature Components

To obtain the variance of ${\tilde{w}}_{k}$ we need to calculate the variance of ${\tilde{c}}_{k}$ and ${\tilde{s}}_{k}$. The variance of the quadrature component is

(A52) $$\mathrm{var}\left({\tilde{s}}_{k}\right)=N\mathrm{var}(|g_{ki}\left|\left|h_{i}^{LIS}\right|\sin\delta_{ki}\right)+\sigma_{3}^{2},$$

where

(A53) $$\begin{array}{c} \mathrm{var}(|g_{ki}|\left|h_{i}^{LIS}\right|\sin\delta_{ki})=\mathrm{var}(|g_{ki}\left|\left|h_{i}^{LIS}\right|\right)\mathrm{var}\left(\sin\delta_{ki}\right)+\ldots \\ \;\mathrm{var}\left(\sin\delta_{ki}\right){\left(E[|g_{ki}|\left|h_{i}^{LIS}\right|]\right)}^{2}+\mathrm{var}(|g_{ki}\left|\left|h_{i}^{LIS}\right|\right){\left(E[\sin\delta_{ki}]\right)}^{2} \end{array}$$

and simplifies to

(A54) $$\begin{array}{c} \mathrm{var}(|g_{ki}|\left|h_{i}^{LIS}\right|\cos\delta_{ki})=\mathrm{var}(|g_{ki}\left|\left|h_{i}^{LIS}\right|\right)\mathrm{var}\left(\cos\delta_{ki}\right)+\ldots \\ \;\mathrm{var}\left(\cos\delta_{ki}\right){\left(E[|g_{ki}|\left|h_{i}^{LIS}\right|]\right)}^{2}+\mathrm{var}(|g_{ki}\left|\left|h_{i}^{LIS}\right|\right){\left(E[\cos\delta_{ki}]\right)}^{2}. \end{array}$$

To compute the variance of the in-phase and quadrature components we need to calculate closed expressions for the trigonometric moments of the Von Mises random variable.

The expected value of the sine of a Von Mises distributed phase error is given as

(A55) $$\begin{array}{ccc} \mathrm{var}(\sin\delta_{ki}) & = & E\left[\sin^{2}\delta_{ki}\right]-{\left(E[\sin\delta_{ki}]\right)}^{2}\\ & = & \frac{1}{2}\left(1-E[\cos2\delta_{ki}]\right)-{\left(E[\sin\delta_{ki}]\right)}^{2}\\ & = & \frac{1}{2}\left(1-\alpha_{2}\right)-\beta_{1}^{2} \end{array}$$

Since $\beta_{1}=0$, so

(A56) $$\mathrm{var}\left(\sin\delta_{ki}\right)=\frac{1}{2}\left(1-\alpha_{2}\right)$$

The expected value of the cosine of a Von Mises variable can be calculated by

(A57) $$\mathrm{var}\left(\cos\delta_{ki}\right)=E\left[\cos^{2}\delta_{ki}\right]-{\left(E[\cos\delta_{ki}]\right)}^{2},$$

or, using a trigonometric substitution,

(A58) $$\mathrm{var}\left(\cos\delta_{ki}\right)=\frac{1}{2}\left(1+E[\cos2\delta_{ki}]\right)-\alpha_{1}^{2},$$

In terms of the the characteristic function, we have

(A59) $$\mathrm{var}\left(\cos\delta_{ki}\right)=\frac{1}{2}\left(1+\alpha_{2}\right)-{\left(\alpha_{1}\right)}^{2}$$

So the variance of ${\tilde{s}}_{k}$ is

(A60) $$\mathrm{var}\left({\tilde{s}}_{k}\right)=N\left[\frac{16-\pi^{2}}{8}\sigma_{12}^{2}\left(1-\alpha_{2}\right)\frac{\pi^{2}}{8}\sigma_{12}^{2}\left(1-\alpha_{2}\right)\right]+\sigma_{3}^{2}$$

and simplifies to

(A61) $$\mathrm{var}\left({\tilde{s}}_{k}\right)=N\left(2\sigma_{12}^{2}\left(1-\alpha_{2}\right)\right)+\sigma_{3}^{2}.$$

The variance of the in-phase fading coefficient will be

(A62) $$\begin{array}{c} \mathrm{var}\left({\tilde{c}}_{k}\right)=N\left[\frac{16-\pi^{2}}{4}\sigma_{12}^{2}\left(\frac{1}{2}\left(1+\alpha_{2}\right)-\alpha_{1}^{2}\right)+\ldots \right.\\ \;\left.\alpha_{1}^{2}\frac{16-\pi^{2}}{4}\sigma_{12}^{2}+\left(\frac{1}{2}\left(1+\alpha_{2}\right)-\alpha_{1}^{2}\right)\frac{\pi^{2}}{4}\sigma_{12}^{2}\right]+\sigma_{3}^{2} \end{array}$$

and simplifies to

(A63) $$\mathrm{var}\left({\tilde{c}}_{k}\right)=\sigma_{3}^{2}+N\left[2\sigma_{12}^{2}\left(1+\alpha_{2}\right)-\frac{\pi^{2}}{4}\alpha_{1}^{2}\sigma_{12}^{2}\right]$$

### Appendix B.3. Mean of ${\left|{\tilde{w}}_{k}\right|}^{2}$

To compute the variance of the total fading coefficient we need the mean value of the squared fading coefficients.

The mean of the squared in-phase component is given as

(A64) $$E\left[{\tilde{c}}_{k}^{2}\right]=\sigma_{3}^{2}+N\left[2\sigma_{12}^{2}\left(1+\alpha_{2}\right)-\frac{\pi^{2}}{4}\alpha_{1}^{2}\sigma_{12}^{2}\right]+{\left(N\frac{\pi }{2}\sigma_{12}\alpha_{1}\right)}^{2}.$$

The expected value of the squared quadrature component can be written as

(A65) $$E\left[{\tilde{s}}_{k}^{2}\right]=\mathrm{var}\left({\tilde{s}}_{k}\right)=N\left(2\sigma_{12}^{2}\left(1-\alpha_{2}\right)\right)+\sigma_{3}^{2}$$

Therefore, the mean squared magnitude of the overall fading with respect to the antenna *k* is given as

(A66) $$E[|{\tilde{w}}_{k}{|}^{2}]=N\left[2\sigma_{12}^{2}\left(1+\alpha_{2}\right)-\frac{\pi^{2}}{4}\alpha_{1}^{2}\sigma_{12}^{2}\right]+{\left(N\frac{\pi }{2}\sigma_{12}\alpha_{1}\right)}^{2}+N\left(2\sigma_{12}^{2}\left(1-\alpha_{2}\right)\right)+2\sigma_{3}^{2}$$

and simplifies to

(A67) $$E[|{\tilde{w}}_{k}{|}^{2}]=4N\left(1+\alpha_{2}\right)\sigma_{12}^{2}+2\sigma_{3}^{2}$$

### Appendix B.4. Correlation between the Fading Coefficients

The real and imaginary parts of the fading coefficients are uncorrelated, however we need to compute the correlation coefficient between the real parts.

The expected value of the product of two different in-phase coefficients can be written as

(A68) $$E\left[{\tilde{c}}_{i}{\tilde{c}}_{k}\right]=E\left[\left(\sum_{l=1}^{N}|g_{il}\left|\right|h_{l}^{LIS}|\cos\delta_{il}+{\tilde{C}}_{BS,i}\right)\times \right.\left.\left(\sum_{m=1}^{N}|g_{km}\left|\right|h_{m}^{LIS}|\cos\delta_{km}+{\tilde{C}}_{BS,k}\right)\right]$$

since all the variables have the same distribution and parameters, therefore if l≠m, so all the summation variables are independent, and in addition $E\left[{\tilde{C}}_{BS,k}\right]\forall k$ and the variables ${\tilde{C}}_{BS,i}$ and ${\tilde{C}}_{BS,k}$ are independent ∀i≠k, therefore

(A69) $$E\left[{\tilde{c}}_{i}{\tilde{c}}_{k}\right]=E\left[{\tilde{C}}_{BS,i}{\tilde{C}}_{BS,k}\right]+\sum_{l=1}^{N}\sum_{m=1}^{N}E\left[|g_{il}\left|\right|g_{km}\left|\right|h_{l}^{LIS}\left|\right|h_{m}^{LIS}|\cos\delta_{il}\cos\delta_{km}\right]$$

and

(A70) $$\begin{array}{c} l\neq m\Rightarrow E\left[|g_{il}\left|\right|g_{km}\left|\right|h_{l}^{LIS}\left|\right|h_{m}^{LIS}|\cos\delta_{il}\cos\delta_{km}\right]=E{\left[|g_{km}|\right]}^{2}E{\left[|h_{m}^{LIS}|\right]}^{2}E{\left[\cos\delta_{km}\right]}^{2}=\\ {\left(\sigma_{1}\sqrt{\frac{\pi }{2}}\right)}^{2}{\left(\sigma_{2}\sqrt{\frac{\pi }{2}}\right)}^{2}\alpha_{1}^{2}=\frac{\pi^{2}}{4}\sigma_{12}^{2}\alpha_{1}^{2} \end{array}$$

and also

(A71) $$\begin{array}{c} l=m,i\neq k\Rightarrow E\left[|g_{il}\left|\right|g_{km}\left|\right|h_{l}^{LIS}\left|\right|h_{m}^{LIS}|\cos\delta_{il}\cos\delta_{km}\right]=E{\left[|g_{km}|\right]}^{2}E\left[|h_{m}^{LIS}{|}^{2}\right]E\left[\cos\delta_{im}\right]E\left[\cos\delta_{km}\right]=\\ \frac{\pi }{2}\sigma_{1}^{2}{\left(2\sigma_{2}\right)}^{2}\alpha_{1}^{2}=\pi \sigma_{12}^{2}\alpha_{1}^{2}, \end{array}$$

(A72) $$\begin{array}{c} l=m,i=k\Rightarrow E\left[|g_{il}\left|\right|g_{km}\left|\right|h_{l}^{LIS}\left|\right|h_{m}^{LIS}|\cos\delta_{il}\cos\delta_{km}\right]=E\left[|g_{km}{|}^{2}\right]E\left[|h_{m}^{LIS}{|}^{2}\right]E\left[\cos^{2}\delta_{km}\right]=\\ 2\sigma_{1}^{2}2\sigma_{2}^{2}\frac{1}{2}\left(1-\alpha_{2}\right) \end{array}$$

therefore

$$E\left[{\tilde{c}}_{i}{\tilde{c}}_{k}\right]=\left\{\begin{array}{cc} N\left(N-1\right)\left(\frac{\pi^{2}}{4}\sigma_{12}^{2}\alpha_{1}^{2}\right)+N\left(\pi \sigma_{12}^{2}\alpha_{1}^{2}\right) & i\neq k\\ N\left(2\sigma_{1}^{2}2\sigma_{2}^{2}\frac{1}{2}\left(1-\alpha_{2}\right)\right)+\sigma_{3}^{2}+\ldots N\left(N-1\right)\left(\frac{\pi^{2}}{4}\sigma_{12}^{2}\alpha_{1}^{2}\right) & i=k \end{array}\right.$$

and the correlation for i≠k is given as

(A73) $$\rho_{{\tilde{c}}_{i}{\tilde{c}}_{k}}=\frac{N\left(N-1\right)\left(\frac{\pi^{2}}{4}\sigma_{12}^{2}\alpha_{1}^{2}\right)+N\left(\pi \sigma_{12}^{2}\alpha_{1}^{2}\right)-{\left(N\frac{\pi }{2}\sigma_{12}\alpha_{1}\right)}^{2}}{\sigma_{3}^{2}+N\left[2\sigma_{12}^{2}\left(1+\alpha_{2}\right)-\frac{\pi^{2}}{4}\alpha_{1}^{2}\sigma_{12}^{2}\right]}$$

### Appendix B.5. Mean of ${\left\|\tilde{w}\right\|}^{2}$

The mean value of the total fading coefficient can be written as

(A74) $$\mu_{{\left\|\tilde{w}\right\|}^{2}}=E\left[{\left\|\tilde{w}\right\|}^{2}\right]=E\left[\sum_{k=1}^{M}{\left|{\tilde{w}}_{k}\right|}^{2}\right].$$

Since the fading coefficients are identically distributed, then

(A75) $$\mu_{{\left\|\tilde{w}\right\|}^{2}}=ME[|{\tilde{w}}_{k}{|}^{2}],$$

and

(A76) $$\mu_{{\left\|\tilde{w}\right\|}^{2}}=4NM\left(1+\alpha_{2}\right)\sigma_{12}^{2}+2M\sigma_{3}^{2}$$

### Appendix B.6. Variance of ${\left\|\tilde{w}\right\|}^{2}$

The variance of the total fading coefficient is given as

(A77) $$\mathrm{var}\left({\left\|\tilde{w}\right\|}^{2}\right)=\mathrm{var}\left(\sum_{k=1}^{M}{\left|{\tilde{w}}_{k}\right|}^{2}\right).$$

Let ${\tilde{Z}}_{k}={\left|{\tilde{w}}_{k}\right|}^{2}$, therefore

(A78) $$\mathrm{var}\left({\left\|\tilde{w}\right\|}^{2}\right)=\mathrm{var}\left(\sum_{i=1}^{M}{\tilde{Z}}_{i}\right)=M\times \mathrm{var}\left({\tilde{Z}}_{i}\right)+M\left(M-1\right)\times \mathrm{cov}\left({\tilde{Z}}_{i},{\tilde{Z}}_{k}\right),$$

Since

(A79) $$\mathrm{cov}\left({\tilde{Z}}_{i},{\tilde{Z}}_{k}\right)=E\left[{\tilde{Z}}_{i}{\tilde{Z}}_{k}\right]-E\left[{\tilde{Z}}_{i}\right]E\left[{\tilde{Z}}_{k}\right],$$

the expected value of the product of the squared magnitude of two diffent fading coefficients is

(A80) $$E\left[{\tilde{Z}}_{i}{\tilde{Z}}_{k}\right]=E\left[{\tilde{c}}_{i}^{2}{\tilde{c}}_{k}^{2}\right]+2E\left[{\tilde{c}}_{i}^{2}{\tilde{s}}_{k}^{2}\right]+E\left[{\tilde{s}}_{i}^{2}{\tilde{s}}_{k}^{2}\right]$$

The term $E[{\tilde{c}}_{i}^{2}{\tilde{c}}_{k}^{2}]$ can be calculated by (A81)

(A81) $$E\left[{\tilde{c}}_{i}^{2}{\tilde{c}}_{k}^{2}\right]=\left\{\begin{array}{cc} 2k_{5}\left(\sigma_{3}^{2}+k_{6}-k_{5}a_{3}\right)+{\left(\sigma_{3}^{2}+k_{6}-k_{5}a_{3}\right)}^{2}+\frac{8}{\pi }k_{5}^{3}a_{3}\left(N+1\right)-3k_{5}^{4}+{\left(\frac{2}{\pi }k_{5}a_{3}\left(N+1\right)-k_{5}^{2}\right)}^{2} & i\neq k\\ k_{5}^{4}+6k_{5}^{2}\left(\sigma_{3}^{2}+k_{6}-k_{5}a_{3}\right)+3{\left(\sigma_{3}^{2}+k_{6}-k_{5}a_{3}\right)}^{2} & i=k \end{array}\right.$$

where $k_{5}=N\frac{\pi }{2}\sigma_{12}\alpha_{1}$, $k_{6}=2N\sigma_{12}^{2}\left(1+\alpha_{2}\right)$ and $a_{3}=\frac{\pi }{2}\sigma_{12}\alpha_{1}$. Since $E[{\tilde{s}}_{k}]=0$, thus the forth order moment of the quadrature component is

(A82) $$E\left[{\tilde{s}}_{k}^{4}\right]=3{\left(\mathrm{var}\left({\tilde{s}}_{k}\right)\right)}^{2}=3{\left(N\left(2\sigma_{12}^{2}\left(1-\alpha_{2}\right)\right)+\sigma_{3}^{2}\right)}^{2}=3{\left(k_{7}+\sigma_{3}^{2}\right)}^{2}$$

where $k_{7}=2N\sigma_{12}^{2}\left(1-\alpha_{2}\right)$.

We need to obtain the term $E\left[{\tilde{c}}_{i}^{2}{\tilde{s}}_{k}^{2}\right]$ without considering the CLT, because the correlation coefficient between $\tilde{c}$ and $\tilde{s}$ is zero. But the terms ${\tilde{c}}_{i}^{2}$ and ${\tilde{s}}_{k}^{2}$ are not independent and the approach that use the integral of the product to obtain the expected value is hard to solve analytically.

Considering the definition, we have that

(A83) $$E\left[{\tilde{c}}_{i}^{2}{\tilde{s}}_{k}^{2}\right]=E\left[{\left(\sum_{l=1}^{N}|g_{il}\left|\right|h_{l}^{LIS}|\cos\delta_{il}+{\tilde{C}}_{BS,i}\right)}^{2}\times \right.\left.{\left(\sum_{m=1}^{N}|g_{km}\left|\right|h_{m}^{LIS}|\sin\delta_{km}+{\tilde{S}}_{BS,k}\right)}^{2}\right]$$

Expanding the power and the product of the two terms we can find the expression (A84). Considering that $E\left[{\tilde{C}}_{BS,i}\right]=0$, $E\left[{\tilde{S}}_{BS,k}\right]=0$, $E\left[{\tilde{S}}_{BS,k}^{2}\right]=E\left[{\tilde{S}}_{BS,k}^{2}\right]=\sigma_{3}^{2}$ so (A84) simplifies to (A85). The three summation terms of (A85) are obtained by (A86), (A87) and (A88) that can be obtained by analyzing the different possible values of the summation indexes that can made the indexed terms dependents or independents.

(A84) $$\begin{array}{c} E\left[{\tilde{c}}_{i}^{2}{\tilde{s}}_{k}^{2}\right]=E\left[{\left(\sum_{l=1}^{N}|g_{il}\left|\right|h_{l}^{LIS}|\cos\delta_{il}\right)}^{2}{\left(\sum_{m=1}^{N}|g_{km}\left|\right|h_{m}^{LIS}|\sin\delta_{km}\right)}^{2}\right]+E\left[{\left(\sum_{l=1}^{N}|g_{il}\left|\right|h_{l}^{LIS}|\cos\delta_{il}\right)}^{2}{\tilde{S}}_{BS,k}^{2}\right]+\\ 2E\left[{\left(\sum_{l=1}^{N}|g_{il}\left|\right|h_{l}^{LIS}|\cos\delta_{il}\right)}^{2}\left(\sum_{m=1}^{N}|g_{km}\left|\right|h_{m}^{LIS}|\sin\delta_{km}\right){\tilde{S}}_{BS,k}\right]+2E\left[\left(\sum_{l=1}^{N}|g_{il}\left|\right|h_{l}^{LIS}|\cos\delta_{il}\right){\tilde{C}}_{BS,i}{\tilde{S}}_{BS,k}^{2}\right]+\\ +2E\left[\left(\sum_{l=1}^{N}|g_{il}\left|\right|h_{l}^{LIS}|\cos\delta_{il}\right){\tilde{C}}_{BS,i}{\left(\sum_{m=1}^{N}|g_{km}\left|\right|h_{m}^{LIS}|\sin\delta_{km}\right)}^{2}\right]+E\left[{\tilde{C}}_{BS,i}^{2}{\left(\sum_{m=1}^{N}|g_{km}\left|\right|h_{m}^{LIS}|\sin\delta_{km}\right)}^{2}\right]+\\ 4E\left[\left(\sum_{l=1}^{N}|g_{il}\left|\right|h_{l}^{LIS}|\cos\delta_{il}\right){\tilde{C}}_{BS,i}\left(\sum_{m=1}^{N}|g_{km}\left|\right|h_{m}^{LIS}|\sin\delta_{km}\right){\tilde{S}}_{BS,k}\right]+E\left[{\tilde{C}}_{BS,i}^{2}{\tilde{S}}_{BS,k}^{2}\right]+\\ 2E\left[{\tilde{C}}_{BS,i}^{2}\left(\sum_{m=1}^{N}|g_{km}\left|\right|h_{m}^{LIS}|\sin\delta_{km}\right){\tilde{S}}_{BS,k}\right] \end{array}$$

(A85) $$\begin{array}{c} E\left[{\tilde{c}}_{i}^{2}{\tilde{s}}_{k}^{2}\right]=E\left[{\left(\sum_{l=1}^{N}|g_{il}\left|\right|h_{l}^{LIS}|\cos\delta_{il}\right)}^{2}{\left(\sum_{m=1}^{N}|g_{km}\left|\right|h_{m}^{LIS}|\sin\delta_{km}\right)}^{2}\right]+\\ \;E\left[{\left(\sum_{l=1}^{N}|g_{il}\left|\right|h_{l}^{LIS}|\cos\delta_{il}\right)}^{2}\right]\sigma_{3}^{2}+E\left[{\left(\sum_{m=1}^{N}|g_{km}\left|\right|h_{m}^{LIS}|\sin\delta_{km}\right)}^{2}\right]\sigma_{3}^{2}+\sigma_{3}^{4} \end{array}$$

(A86) $$\begin{array}{c} E\left[{\left(\sum_{l=1}^{N}|g_{il}\left|\right|h_{l}^{LIS}|\cos\delta_{il}\right)}^{2}\right]=E\left[\sum_{l=1}^{N}\sum_{t=1}^{N}|g_{il}\left||\right|g_{it}\left|\right|h_{l}^{LIS}\left|\right|h_{t}^{LIS}|\cos\delta_{il}\cos\delta_{it}\right]=\\ \;2N\sigma_{12}^{2}\left(1+\alpha_{2}\right)+\left(N^{2}-N\right){\left(\frac{\pi }{2}\right)}^{2}\sigma_{12}^{2}\alpha_{1}^{2} \end{array}$$

(A87) $$\begin{array}{c} E\left[{\left(\sum_{m=1}^{N}|g_{km}\left|\right|h_{m}^{LIS}|\sin\delta_{km}\right)}^{2}\right]=E\left[\sum_{d=1}^{N}\sum_{m=1}^{N}\left|\right|g_{kd}\left|\right|g_{km}\left|\right|h_{d}^{LIS}\left|\right|h_{m}^{LIS}|\sin\delta_{kd}\sin\delta_{km}\right]=2N\sigma_{12}^{2}\left(1-\alpha_{2}\right) \end{array}$$

(A88) $$\begin{array}{c} E\left[{\left(\sum_{l=1}^{N}|g_{il}\left|\right|h_{l}^{LIS}|\cos\delta_{il}\right)}^{2}{\left(\sum_{m=1}^{N}|g_{km}\left|\right|h_{m}^{LIS}|\sin\delta_{km}\right)}^{2}\right]=\\ \;E\left[\sum_{l=1}^{N}\sum_{t=1}^{N}\sum_{d=1}^{N}\sum_{m=1}^{N}|g_{il}\left||\right|g_{it}\left||\right|g_{kd}\left|\right|g_{km}\left|\right|h_{l}^{LIS}\left|\right|h_{t}^{LIS}\left|\right|h_{d}^{LIS}\left|\right|h_{m}^{LIS}|\cos\delta_{il}\cos\delta_{it}\sin\delta_{kd}\sin\delta_{km}\right] \end{array}$$

$E\left[{\tilde{c}}_{i}^{2}{\tilde{s}}_{k}^{2}\right]=$

$$\left\{\begin{array}{cc} \left(N-1\right)k_{10}+3\left(N^{2}-N\right)k_{9}+2k_{10}+\left(N^{3}-3N^{2}+2N\right)k_{9}+\left(k_{6}+\frac{\left(N-1\right)}{N}k_{5}+k_{7}\right)\sigma_{3}^{2}+\sigma_{3}^{4} & i\neq k\\ \left(N-1\right)k_{8}+\frac{9}{2}\left(N^{2}-N\right)k_{11}+4k_{10}+\left(N^{3}-3N^{2}+2N\right)k_{9}+\left(k_{6}+\frac{\left(N-1\right)}{N}k_{5}+k_{7}\right)\sigma_{3}^{2}+\sigma_{3}^{4} & i=k \end{array}\right.$$

where

(A89) $$k_{8}=2N\sigma_{12}^{4}\left(1-\alpha_{4}\right),\;k_{9}=2{\left(\frac{\pi }{2}\right)}^{2}\sigma_{12}^{4}\alpha_{1}^{2}\left(1-\alpha_{2}\right),\;k_{10}=4N\sigma_{12}^{4}\left(1-\alpha_{2}^{2}\right),\;k_{11}={\left(\frac{\pi }{2}\right)}^{2}\sigma_{12}^{4}\alpha_{1}\left(\alpha_{1}-\alpha_{3}\right)$$

$E[{\tilde{Z}}_{i}{\tilde{Z}}_{k}]=$

(A90) $$\left\{\begin{array}{cc} 2k_{5}\left(\sigma_{3}^{2}+k_{6}-k_{5}a_{3}\right)+{\left(\sigma_{3}^{2}+k_{6}-k_{5}a_{3}\right)}^{2}+\frac{8}{\pi }k_{5}^{3}a_{3}\left(N+1\right)-3k_{5}^{4}+{\left(\frac{2}{\pi }k_{5}a_{3}\left(N+1\right)-k_{5}^{2}\right)}^{2}+\\ 2\left[\left(N-1\right)k_{10}+3\left(N^{2}-N\right)k_{9}+2k_{10}+\left(N^{3}-3N^{2}+2N\right)k_{9}+\left(k_{6}+\frac{\left(N-1\right)}{N}k_{5}+k_{7}\right)\sigma_{3}^{2}+\sigma_{3}^{4}\right]+\\ 2\left(\sigma_{3}^{2}+k_{6}-k_{5}a_{3}+k_{5}^{2}\right)\left(k_{7}+\sigma_{3}^{2}\right)+{\left(k_{7}+\sigma_{3}^{2}\right)}^{2} & i\neq k\\ k_{5}^{4}+6k_{5}^{2}\left(\sigma_{3}^{2}+k_{6}-k_{5}a_{3}\right)+3{\left(\sigma_{3}^{2}+k_{6}-k_{5}a_{3}\right)}^{2}+2\left(\sigma_{3}^{2}+k_{6}-k_{5}a_{3}+k_{5}^{2}\right)\left(k_{7}+\sigma_{3}^{2}\right)+\\ 2\left[\left(N-1\right)k_{8}+\frac{9}{2}\left(N^{2}-N\right)k_{11}+4k_{10}+\left(N^{3}-3N^{2}+2N\right)k_{9}+\left(k_{6}+\frac{\left(N-1\right)}{N}k_{5}+k_{7}\right)\sigma_{3}^{2}+\sigma_{3}^{4}\right]+\\ 3{\left(k_{7}+\sigma_{3}^{2}\right)}^{2} & i=k \end{array}\right.$$

Thus the covariance $\mathrm{cov}({\tilde{Z}}_{i},{\tilde{Z}}_{k})$ can be obtained by (A79), where $E\left[{\tilde{Z}}_{i}{\tilde{Z}}_{k}\right]$ is given by (A90), remember that $E\left[{\tilde{Z}}_{i}\right]=E\left[{\tilde{Z}}_{k}\right]$ and $E\left[{\tilde{Z}}_{k}\right]=E\left[|{\tilde{w}}_{k}{|}^{2}\right]$ that can be calculated by (A67), also consider that $\mathrm{var}\left({\tilde{Z}}_{k}\right)=\mathrm{cov}\left({\tilde{Z}}_{k},{\tilde{Z}}_{k}\right)$ and the variance of the overall fading coefficient is given by (A91).

(A91) $$\begin{array}{c} \sigma_{\left\|{\tilde{w}}^{2}\right\|}^{2}=\mathrm{var}\left(\left\|{\tilde{w}}^{2}\right\|\right)=\\ \;M\left(M-1\right)\left[2k_{5}\left(\sigma_{3}^{2}+k_{6}-k_{5}a_{3}\right)+{\left(\sigma_{3}^{2}+k_{6}-k_{5}a_{3}\right)}^{2}+\frac{8}{\pi }k_{5}^{3}a_{3}\left(N+1\right)-3k_{5}^{4}+{\left(\frac{2}{\pi }k_{5}a_{3}\left(N+1\right)-k_{5}^{2}\right)}^{2}+\right.\\ \;\left.2\left(\left(N-1\right)k_{10}+3\left(N^{2}-N\right)k_{9}+2k_{10}+\left(N^{3}-3N^{2}+2N\right)k_{9}+\left(k_{6}+\frac{\left(N-1\right)}{N}k_{5}+k_{7}\right)\sigma_{3}^{2}+\sigma_{3}^{4}\right)+\right.\\ \;\left.2\left(\sigma_{3}^{2}+k_{6}-k_{5}a_{3}+k_{5}^{2}\right)\left(k_{7}+\sigma_{3}^{2}\right)+{\left(k_{7}+\sigma_{3}^{2}\right)}^{2}-{\left(2k_{6}+2\sigma_{3}^{2}\right)}^{2}\right]+\\ \;M\left[k_{5}^{4}+6k_{5}^{2}\left(\sigma_{3}^{2}+k_{6}-k_{5}a_{3}\right)+3{\left(\sigma_{3}^{2}+k_{6}-k_{5}a_{3}\right)}^{2}+2\left(\sigma_{3}^{2}+k_{6}-k_{5}a_{3}+k_{5}^{2}\right)\left(k_{7}+\sigma_{3}^{2}\right)+\right.\\ \;2\left(\left(N-1\right)k_{8}+\frac{9}{2}\left(N^{2}-N\right)k_{11}+4k_{10}+\left(N^{3}-3N^{2}+2N\right)k_{9}+\left(k_{6}+\frac{\left(N-1\right)}{N}k_{5}+k_{7}\right)\sigma_{3}^{2}+\sigma_{3}^{4}\right)+\\ \;\left.3{\left(k_{7}+\sigma_{3}^{2}\right)}^{2}-{\left(2k_{6}+2\sigma_{3}^{2}\right)}^{2}\right] \end{array}$$

## Appendix C. Trigonometric Moments of a Von Mises Random Variable

Since the Von Mises distribution is symmetric about zero, therefore the expected values of odd functions applied to a Von Mises distributed random variable will be zero, therefore

(A92) $$\forall n,m\in Z\;E[\sin^{2n+1}\delta \cos^{m}\delta ]=0,$$

on the other hand, the expected value of a power of even trigonometric functions must be expanded in trigonometric Fourier series, or simply transformed in a superposition of cosine and sine functions using trigonometric transformations to allow us to use the characteristic function definition.

Since $E\left[\cos p\delta \right]=\alpha_{p}$, we have that

(A93) $$E\left[\cos^{2}\delta \right]=E\left[\frac{1}{2}\left(1+\cos2\delta \right)\right]=\frac{1}{2}\left(1+\alpha_{2}\right),$$

(A94) $$E\left[\cos^{3}\delta \right]=E\left[\frac{1}{4}\left(3\cos\delta +\cos3\delta \right)\right]=\frac{1}{4}\left(3\alpha_{1}+\alpha_{3}\right),$$

and

(A95) $$E\left[\cos^{4}\delta \right]=E\left[\frac{1}{8}\left(3+4\cos2\delta +\cos4\delta \right)\right]=\frac{1}{8}\left(3+4\alpha_{2}+\alpha_{4}\right).$$

We can write the power of a sine function in terms of the cosine function by applying the algebraic transformation $\sin^{2}\delta =1-\cos^{2}\delta$, and this substitution is useful only when the power is even. The expected value is zero for every odd power of sinδ.
